# A Holocene Sediment Record of Phosphorus Accumulation in Shallow Lake Harris, Florida (USA) Offers New Perspectives on Recent Cultural Eutrophication

**DOI:** 10.1371/journal.pone.0147331

**Published:** 2016-01-20

**Authors:** William F. Kenney, Mark Brenner, Jason H. Curtis, T. Elliott Arnold, Claire L. Schelske

**Affiliations:** 1 Land Use and Environmental Change Institute, University of Florida, Gainesville, Florida, United States of America, 32611; 2 Department of Geological Sciences, University of Florida, Gainesville, Florida, United States of America, 32611; Institute of Tibetan Plateau Research, CHINA

## Abstract

We studied a complete Holocene sediment record from shallow (z_max_ = 9.7 m) Lake Harris, Florida (USA) to infer the historical development of the lake and its current eutrophic status. We used ^210^Pb and ^14^C to date the 5.9-m sediment sequence (core LH-6-13) and determined accumulation rates for bulk sediment, organic matter, calcium carbonate, phosphorus fractions and biogenic silica fractions. The chronology of changes in sediment characteristics for LH-6-13 is consistent with the general paleoenvironmental framework established by core studies from other Florida lakes. Lake Harris began to fill with water in the early Holocene, ca. 10,680 cal a BP. A shift from carbonate-dominated to organic-rich sediments ca. 5,540 cal a BP corresponds to a transition to wetter climate in the middle Holocene. A rapid increase in diatom biogenic silica concentrations and accumulation rates ca. 2,600 cal a BP signals that the lake had deepened to its modern limnetic state. In LH-6-13, an up-core decrease in rates of accumulation for several sediment variables indicates time-course oligotrophication of the lake through the Holocene. In near-surface sediments, abrupt increases in the accumulation rates of these same variables indicate progressive cultural eutrophication after ca. AD 1900. Comparison of the modern state of Lake Harris to its condition 50–100 years ago provides a measure of the impact of recent cultural eutrophication. Because the pre-disturbance trajectory of this lake was one of oligotrophication, the true impact of cultural eutrophication is even greater than what is inferred from the changes over the past century.

## Introduction

Widespread eutrophication of North American lakes is documented by 50 years of limnological research and lake management efforts [1]. For some lakes, it has been argued that recent eutrophication resulted from time-course nutrient enrichment as a consequence of primary succession, but this concept was not supported by a study of boreal lakes of different ages [2] or paleolimnological investigations in temperate water bodies [3]. In subtropical Florida, USA, the presence of phosphorus (P)-rich geologic deposits led some to consider these lakes to be naturally productive [4] or historically eutrophic [5]. Because they may be naturally productive, it is possible that subtropical Florida lakes show a pattern of primary succession with respect to eutrophication that is different from that found in boreal or temperate lakes of North America.

Many sediment records from Florida lakes show evidence of recent eutrophication [6–11], but these studies focused on short cores (<1.5 m) and relatively recent sediments (post-1900). Smith et al. [1] questioned whether such short cores penetrate deep enough into the sediment to sample pre-disturbance conditions effectively in many North American lakes. We propose that a long core from shallow (z_max_ = 9.6 m), sub-tropical Lake Harris, FL, which contains a complete Holocene sediment record, might be useful for distinguishing the role of cultural eutrophication from eutrophication that resulted from primary succession.

In Florida lakes, both pollen and diatom stratigraphies from Holocene sediment records have improved our understanding of paleoclimate and lake ontogeny. These records provide paleoecological time markers that can help validate the chronology of changes in sediment characteristics found in our study and in future investigations. Diatoms in a sediment core from shallow Lake Apopka, Florida indicated that the lake began to fill early in the Holocene, *ca*. 8,500 ^14^C a BP (i.e. radiocarbon years before present, where present = 1950) [12]. Pollen stratigraphies from several Florida lake cores showed a succession from oak to pine *ca*. 5,500 cal a BP, indicating a progression to a wetter climate [13–19]. This pollen-inferred shift to wetter climate in the middle Holocene precedes a shift in diatom taxa found in spring-fed Lake Apopka. In Lake Apopka, the composition of the diatom assemblage changed *ca*. 2,800 a BP. The relative proportion of benthic diatoms decreased to <50% and the relative proportion of planktonic diatoms increased to >50% [12]. This shift in the diatom assemblage indicates the lake had deepened to its modern limnetic state by that time [12]. Given the paleoecological framework apparent from previous studies of Holocene sediment sequences from Florida lakes, we expected that the sediment record from nearby Lake Harris would show that the lake began to fill in the early Holocene, that there had been a shift to wetter climate *ca*. 5,500 a BP and that the lake deepened to its modern condition *ca*. 2,800 a BP.

We used geochemical analyses, ^210^Pb dating and ^14^C dating of a complete Holocene sediment record from shallow Lake Harris, FL to investigate the historical development of eutrophic conditions in the lake. We evaluated concentrations and accumulation rates of organic matter (OM) as proxies for net primary productivity, and three forms of phosphorus, i.e. water-soluble P (H_2_O-P), heat-extractable P (HE-P) and total P (TP), as proxies for lake P enrichment. We evaluated concentrations and accumulation rates of carbonate (as CaCO_3_) as proxies for groundwater inputs, and sponge spicule biogenic silica (BSi_sponges_) and diatom biogenic silica (BSi_diatoms_) as proxies for macrophyte abundance and phytoplankton abundance, respectively [8], [9]. We used these sediment variables to demonstrate that the stratigraphic sequence of sediment characteristics in Lake Harris is consistent with existing Holocene records from other Florida lakes, and to test whether the lake’s Holocene sediment sequence shows a time-course pattern of eutrophication.

### Study site

**Fig 1:**
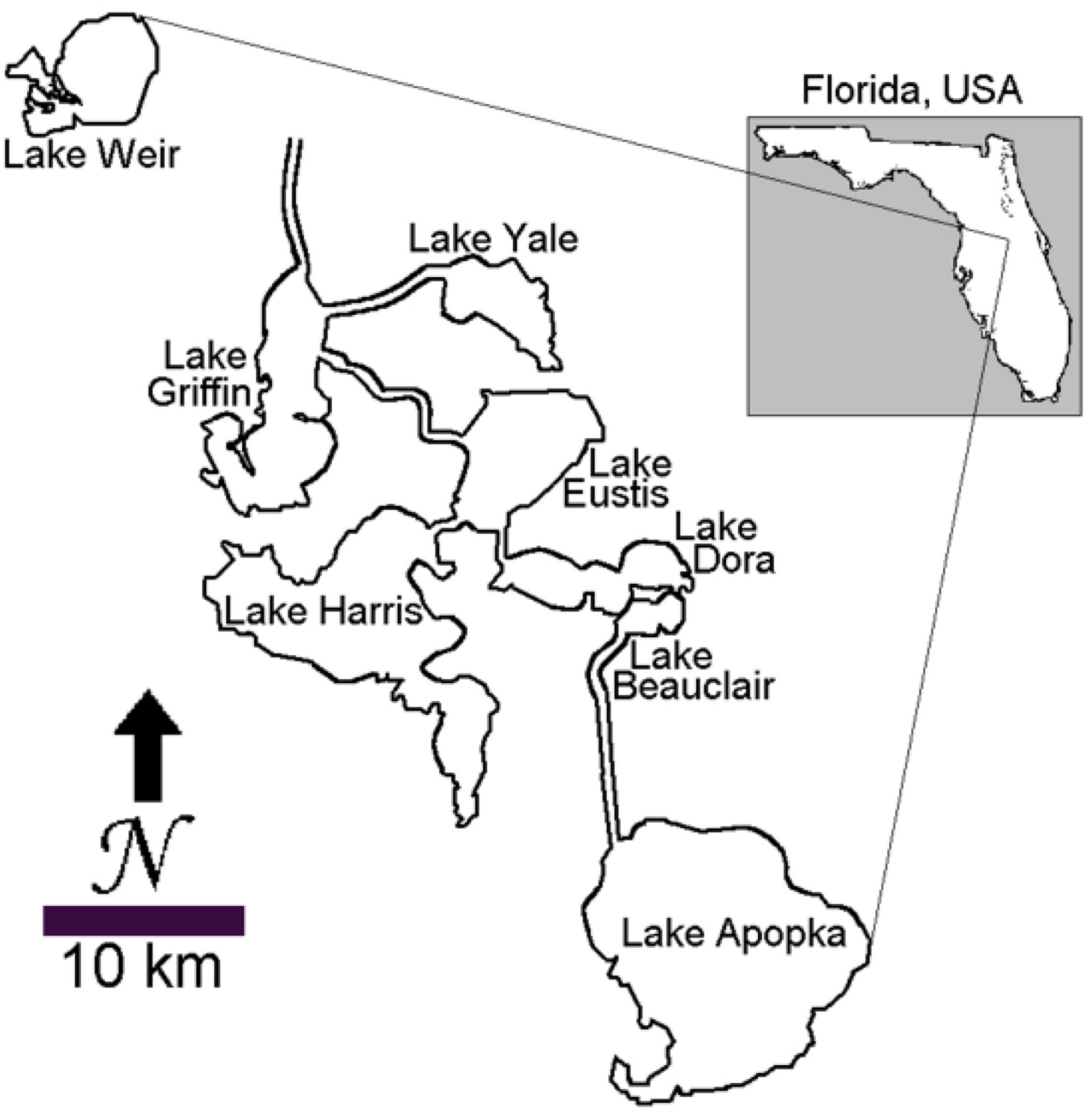
Map of study area. Map showing the location of Lake Harris within the Harris Chain of Lakes. Widths of canals and rivers are exaggerated. Reproduced from [9] with permission from Springer, license number 3761470710006.

Lake Harris (Fig 1) is a subtropical, eutrophic, shallow water body located 50 km NW of Orlando, FL, USA [4]. The lake has a surface area of 75 km^2^, a mean depth of 3.5 m and is considered naturally productive [4]. In a previous study, short (<1.5 m) sediment/water interface cores from the lake showed progressive increases in TP concentration and greater phytoplankton contributions to sediment OM since about AD 1950 [9]. These changes correspond temporally to a 6-fold increase in the population of local residents between 1950 and 2000 [20]. Soft sediments cover more than 95% of the bottom of Lake Harris [21]. The average thickness of soft sediments is 2.6 m and the maximum thickness is 8.8 m [21]. We selected a coring location (LH-6, [9]) with 5.9 m of soft sediment. Because the soft sediment thickness at LH-6 falls within the upper quintile of soft sediment thickness throughout Lake Harris, we expected that a core from the site would be representative of the lake’s environmental history.

## Methods

### Sediment sampling

A 1.2-m sediment/water interface core was collected with a piston corer [22] on 9 April 2013. The sediment/water interface core was extruded vertically and sectioned at 4-cm intervals in the field. Those samples were stored in low-density polyethylene cups and placed in ice chests for transport to the laboratory. Deeper in the sediment, we collected six one-meter, overlapping cores with a locking piston corer that uses plastic core barrels. We obtained a complete, 5.9-m profile of soft sediment and the deeper core sections were transported to the laboratory in the plastic core tubes. Lake Harris is a public resource with free access at all times, thus special permission to collect sediment cores was not required. In the laboratory, the 1-m core sections were split in half length-wise. One half was sectioned at 4-cm intervals for immediate analyses, whereas the other half was stored at 4°C to be used for charcoal collection and future analyses. Wet subsamples were removed from each section for analysis of H_2_O-P and HE-P. Sediment sections were frozen, freeze-dried and ground to a fine powder in the laboratory in preparation for the remaining analyses. This field study did not involve endangered or protected species.

### Sediment chronology

The sediment/water interface core was dated by ^210^Pb. Radiometric measurements (^210^Pb, ^226^Ra and ^137^Cs) were made using low-background gamma counting with well-type intrinsic germanium detectors [23], [24]. Sediment ages were calculated using the constant rate of supply (CRS) model [25], [26]. Age errors were propagated using first-order approximations and calculated according to Binford [27]. Four charcoal samples, from 140, 386, 504 and 579 cm depth, and three bulk-sediment samples, from 78, 140 and 198 cm depth were AMS ^14^C-dated at Beta Analytic Laboratory in Miami, Florida, USA. The paired charcoal (^14^C_charcoal_) and bulk (^14^C_bulk_) samples from 140 cm depth yielded an age difference of 247 years. We attributed the greater age of the bulk sample to hard-water-lake error and used the age discrepancy to “correct” the ^14^C_bulk_ dates on samples from 78 and 198 cm, assuming the magnitude of hard-water-lake error was constant through time.

### Sediment characterization

Sediment bulk density (g_dry_ cm^-3^_wet_) was determined from the proportion of dry matter in wet sediment and proportions of inorganic and organic matter in dry sediment, using the equation of Binford [27]. Organic matter content was determined by weight loss on ignition after combustion at 550°C for 2 h. Analytical procedures for H_2_O-P and HE-P are presented in Kenney et al. [28] and TP was measured according to Schelske et al. [29]. We used time-course leaching [30] to determine BSi_Diatoms_ and BSi_Sponges_ and the sum of those components is BSi_total_. Total inorganic carbon was measured coulometrically [31] using a UIC (Coulometrics) 5011 CO_2_ coulometer coupled with an AutoMate automated carbonate preparation device (AutoMateFX.com). Replicate analyses were run on 10% of all samples and relative percent differences for sample replicates were <10% for bulk density, OM, BSi_Diatoms_, BSi_Sponges_, H_2_O-P, HE-P, and <5% for TP and CaCO_3_.

## Results

### Sediment chronology

**Fig 2:**
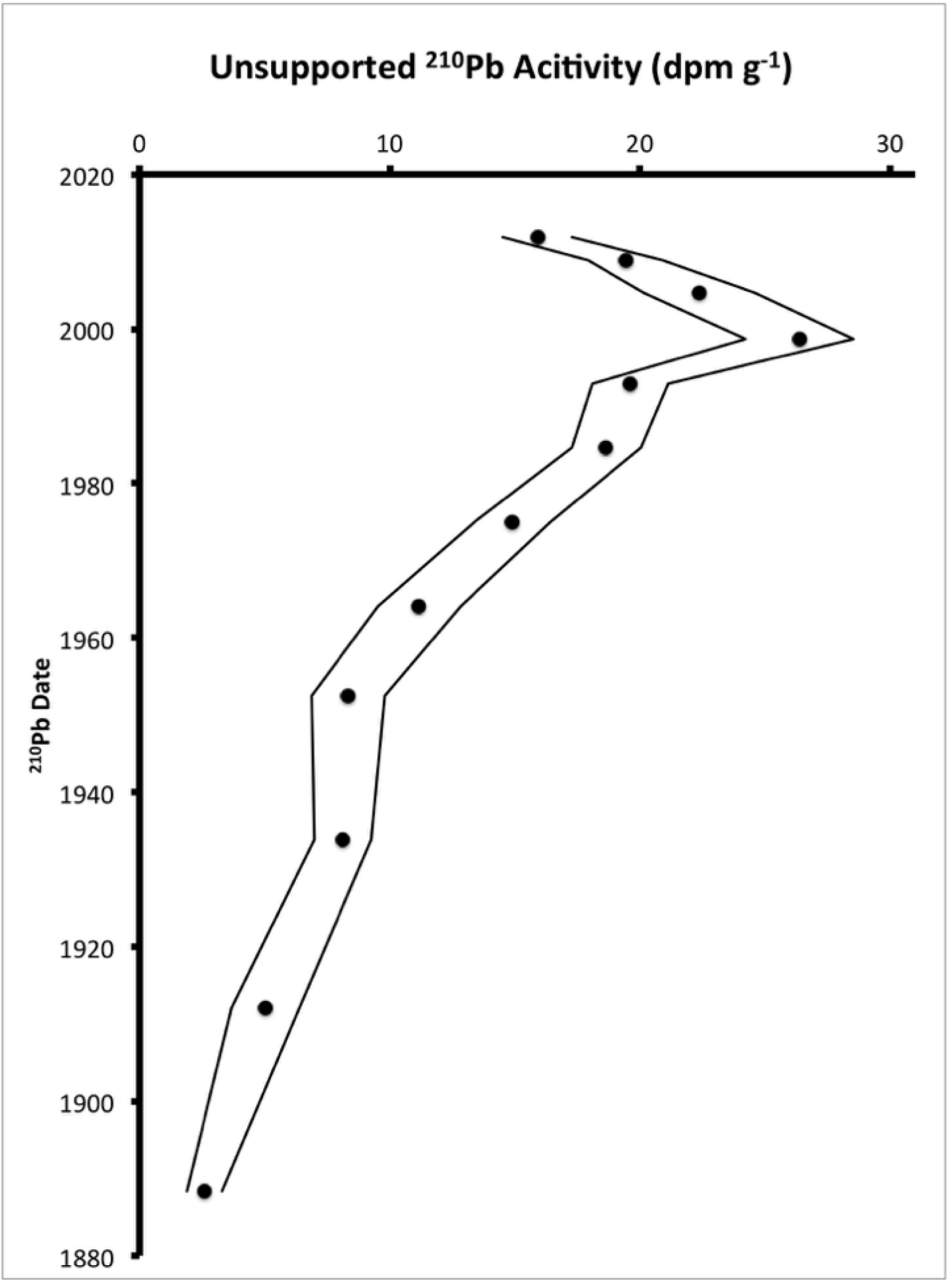
^210^Pb Data. Unsupported ^210^Pb activity (i.e. total ^210^Pb– ^226^Ra) versus ^210^Pb date for Lake Harris sediment core LH-6-13 (closed circles). Error bars for the activity (solid lines) were calculated from the square root of net counts.

**Fig 3:**
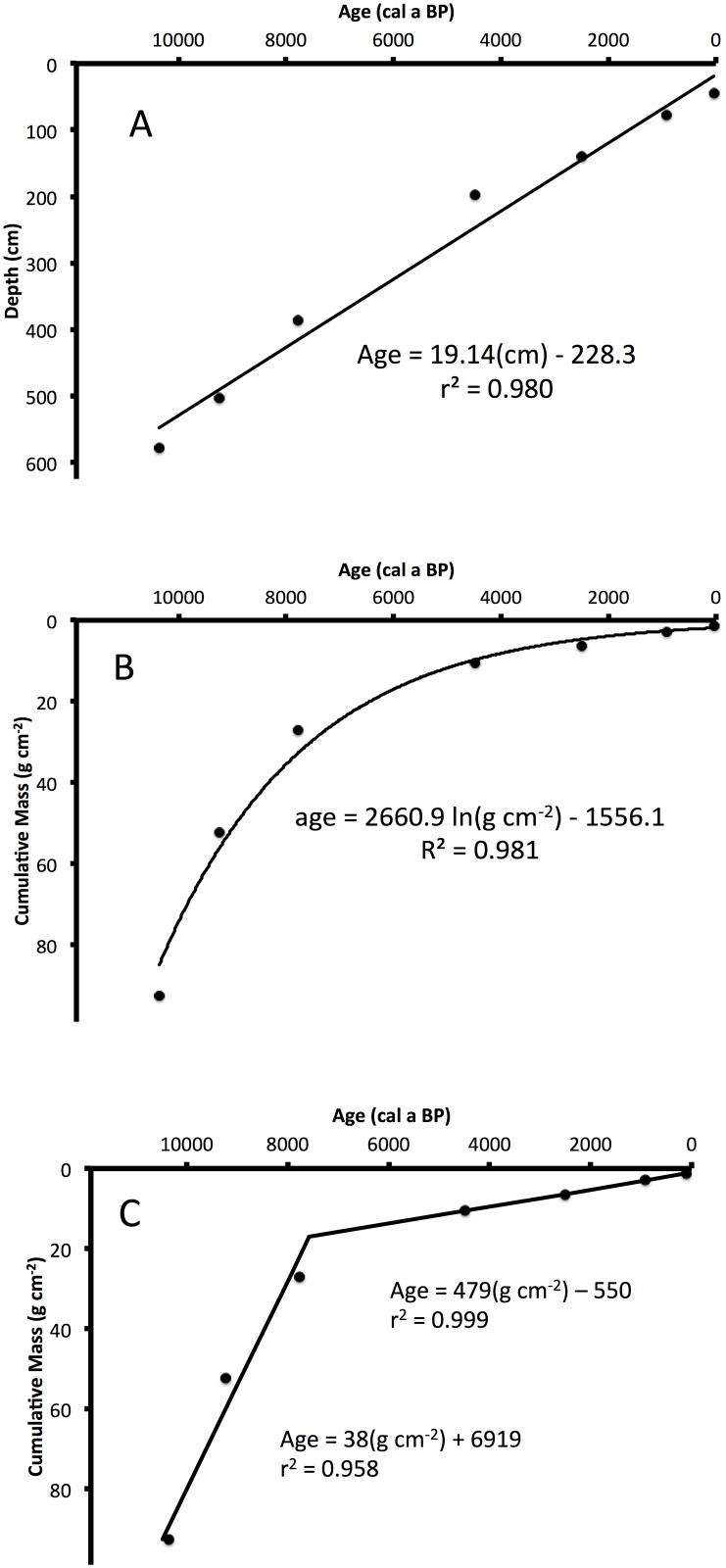
Age-Depth Models. (A) Linear, (B) exponential and (C) two-stage linear chronological models are presented for Lake Harris core LH-6-13, with model formulae and model performance (r^2^).

The combined ^210^Pb and ^14^C dates provide a reliable chronology for LH-6-13 (Table 1). Unsupported ^210^Pb activity decreased with depth in the upper 44 cm of sediment (Fig 2) and unsupported ^210^Pb activities from LH-6-13 are consistent with ^210^Pb activities measured in a core collected at the same location in 1999 [32]. Lead-210 dates provide a high-resolution age-depth relation for recent sediments. The ^210^Pb date at 44 cm depth was ~1911, equivalent to ~40 a BP (P = 1950), and was used in conjunction with six ^14^C ages to model the entire Holocene chronology. Carbon-14 dates show a linear age-depth relationship (r^2^ = 0.98, Fig 3a) and a logarithmic age-cumulative mass relationship (R^2^ = 0.98, Fig 3b). A two-component linear model of age versus cumulative mass had an inflection point at ~284 cm, corresponding to an age of *ca*. 7,560 cal a BP (r^2^_upper section_ = 0.999, r^2^_lower section_ = 0.958, Fig 3c). Another chronological model assumed constant mass sedimentation rate (MSR) between samples with measured ages. To estimate dates for depths where critical changes in sediment characteristics occur (critical depths), we used the average of the three most similar age predictions from four models (Table 2).

**Table 1:** Core Chronological Data.

		^210^Pb-Age	Calibrated	Calibrated		Model Input	2σ Age	Model Input	Model Input
AMS-Lab	Depth		^14^C_charcoal_-Age	^14^C_bulk_-Age	Correction	Age	Error	Depth	Cumulative Mass
Sample ID	cm	a	a	a	a	a	A	cm	g cm^-2^
	0–4	1.5					1.6	4	0.057
	4–8	4.4					1.6	8	0.143
	8–12	8.6					1.7	12	0.237
	12–16	14.7					1.9	16	0.336
	16–20	20.4					2.0	20	0.442
	20–24	28.7					2.4	24	0.569
	24–28	38.3					2.8	28	0.725
	28–32	49.2					3.3	32	0.881
	32–36	60.9					3.9	36	1.043
	36–40	79.5					5.9	40	1.206
	40–44	101.2				39	7.8	44	1.378
394911	76–80			1265–1065	-247	922	62	78	2.857
391984	134–145		2695–2635			2498	110	140	6.426
			2615–2595						
			2500–2350						
394912	136–140			2760–2720		**2745**	12		
394913	196–200			4835–4785	-247	4489	76	198	10.512
				4765–4615					
396995	374–398		7840–7690			7770	42	386	27.028
396996	498–510		9400–9360			9235	69	504	52.297
			9315–9130						
361548	575–583		10500–10250			10362	69	579	92.638

Chronological data for core LH-6-13. To correct for hard-water-lake error, we subtracted 247 years from the calibrated age for bulk sediment samples 394911 and 394913. The hard-water-lake correction was determined by comparing the ^14^C ages of a bulk sediment sample (394912) and a charcoal sample (391984) collected at similar depths in the core.

**Table 2:** Modeled Ages for Critical Depths.

Critical Depths		Base of Core	Unique Sand Layer	H_2_O-P Maximum	No CaCO_3_	Diatom Transition-lower	Diatom Transition-upper
Depth (cm)		590	354	236	232	144	132
Cumulative Mass (g cm^-2^)		100.1	21.9	13.9	13.5	6.7	5.8
f(cm) = age	Linear	10699	6658	5444	5364	*3501	*3116
f(g cm^-2^) = age	Exponential	10765	*7761	6104	5906	2656	2224
f(g cm^-2^) = age	2-stage linear	*11064	6547	*4289	*4212	2528	2298
Constant MSR (interval)		10569	6753	5077	5077	2614	2216
Mean (age)	Best three	10678	6653	5542	5449	2599	2246
2σ Age Error		199	206	1041	842	131	90

Modeled ages for critical depths in core LH-6-13. For each critical depth, we estimated the age as the average of the three most similar model outputs of the four models created to establish the chronology of LH-6-13. A “*” indicates that the model output was not used to estimate the age for each critical depth.

### Sediment characteristics

**Fig 4:**
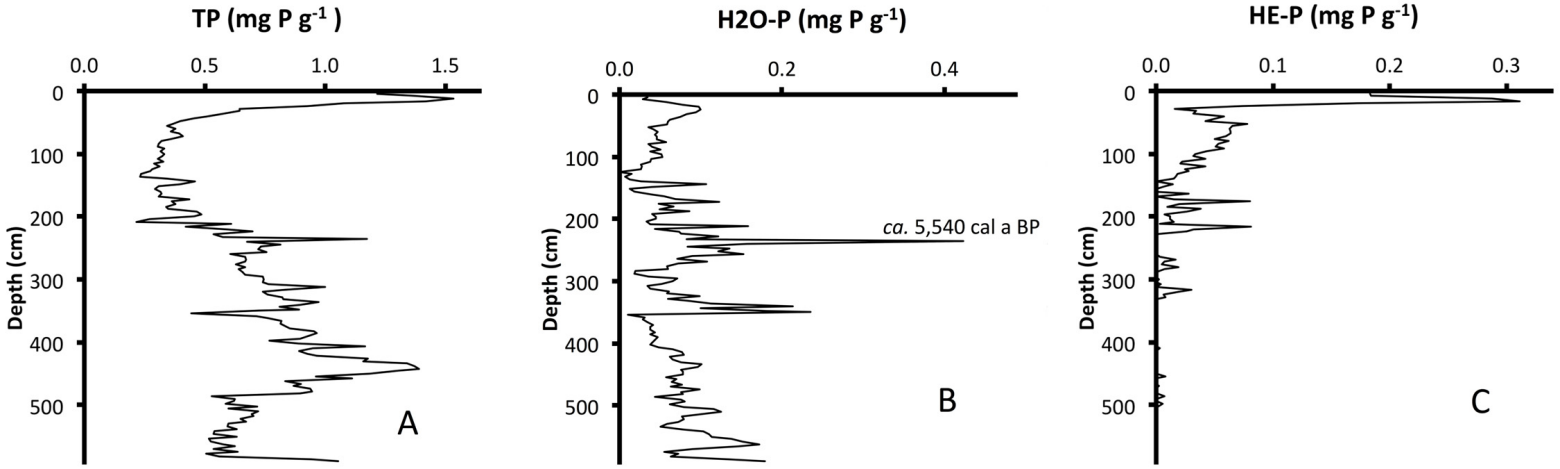
Phosphorus Stratigraphy. Concentrations of (A) total phosphorus (TP), (B) water-soluble phosphorus (H_2_O-P) and (C) heat-extractable phosphorus (HE-P) versus depth in LH-6-13. The modeled date for the maximum concentration of H_2_O-P at 236 cm is *ca*. 5,540 cal a BP.

**Fig 5:**
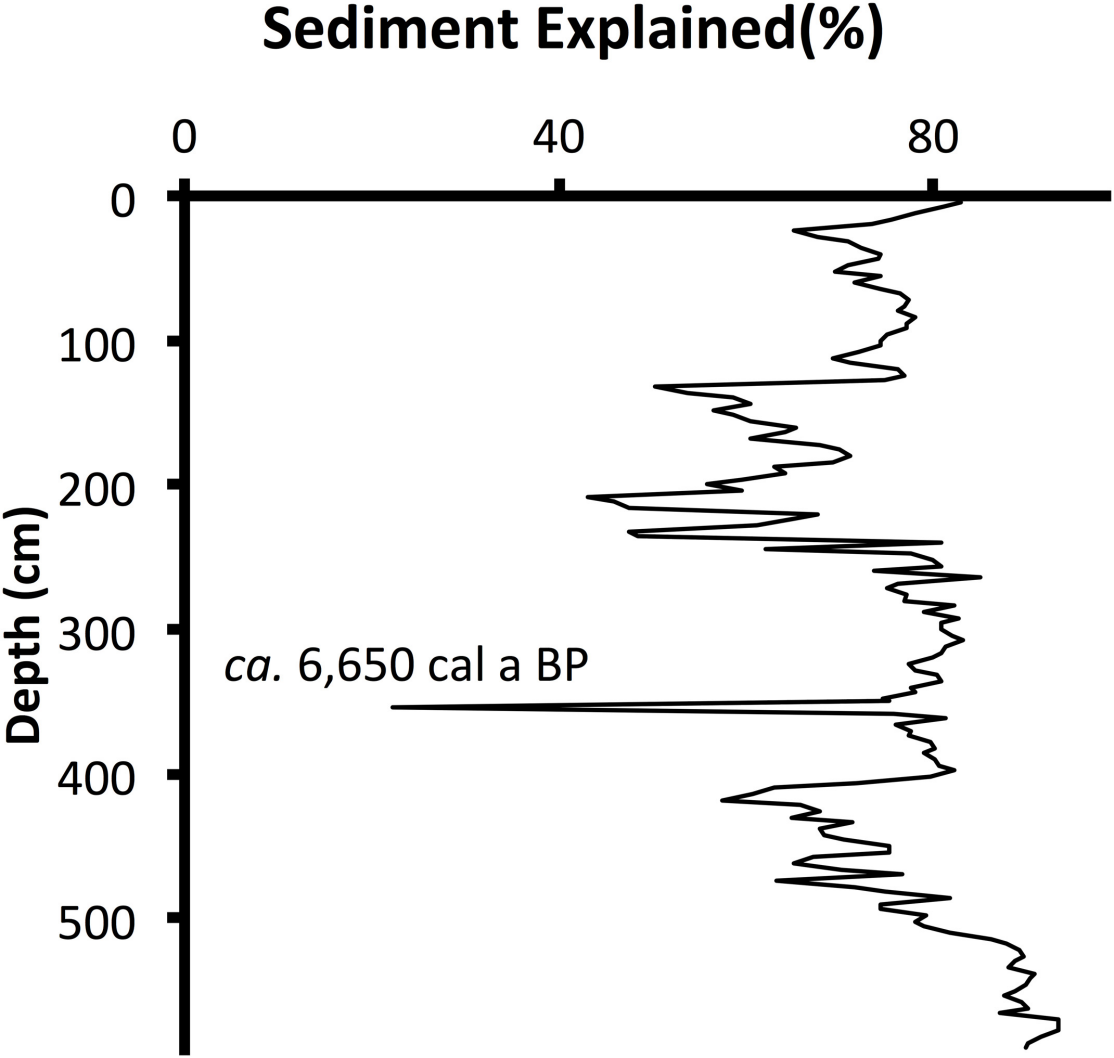
Unique Sand Layer. Percent sediment mass explained by the sum of organic matter, CaCO_3_ and biogenic silica (sponge spicules + diatoms) versus depth in core LH-6-13. These data helped identify the sand layer at 354 cm, which has a modeled age of *ca*. 6,650 cal a BP.

**Fig 6:**
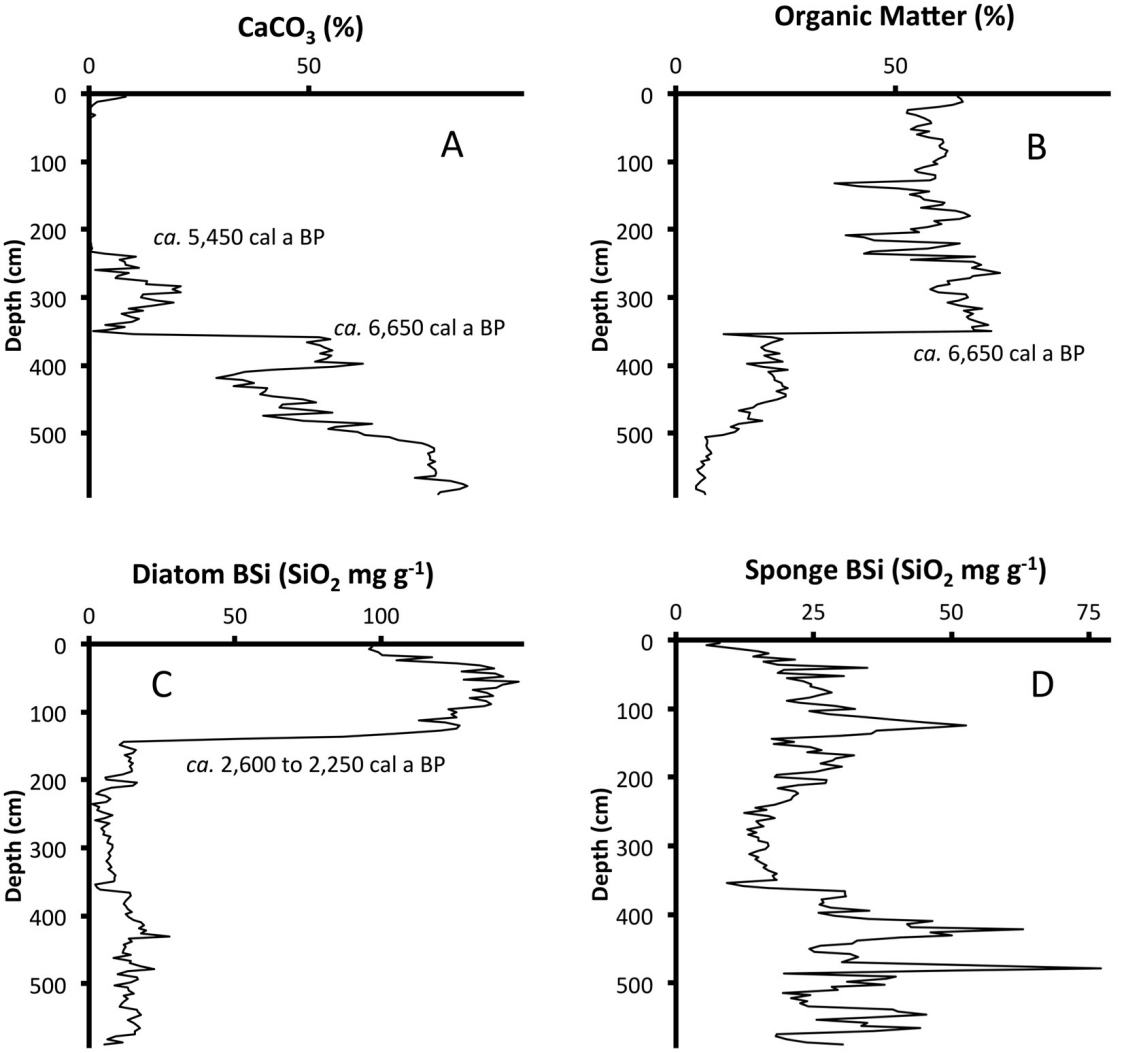
Stratigraphies of Carbonate, Organic Matter and Biogenic Silica. Concentrations of (A) CaCO_3_, (B) organic matter (OM), (C) diatom biogenic silica and (D) sponge spicule biogenic silica versus depth in core LH-6-13. Biogenic silica concentrations are reported as SiO_2_. The sediment shifted from CaCO_3_-dominated (> 50%) to OM-dominated (>50%) at 354 cm (*ca*. 6,650 cal a BP). Above 232 cm (*ca*. 5,440 cal a BP), CaCO_3_ concentrations never exceed 0.2%, except in surface sediments, where they are < 10%. From 140 cm to 132 cm (*ca*. 2,600 to *ca*. 2,250 cal a BP), diatom biogenic silica increased to >10% of sediment mass.

Characteristics of the recent sediments show a pattern of progressive eutrophication that contrasts with the apparent time-course oligotrophication indicated by the characteristics in the deeper Holocene sediments. In general, TP concentrations decreased up-core through the Holocene until *ca*. AD 1900 (44 cm). Above 44 cm, TP concentrations increased up-core to their maximum value (Fig 4a). The chronology of stratigraphic changes in sediment characteristics for LH-6-13 was consistent with paleo-ecological markers established in previous studies of Florida lakes [12–19]. There is a unique sand layer at 354 cm (*ca*. 6,650 ± 210 cal a BP, Fig 5) and a shift from carbonate-rich sediments (Fig 6a) to organic-rich sediments (Fig 6b) identifies the oldest stratigraphic change in sediment characteristics. Above the sand layer, the carbonate content of sediments decreased up-core until carbonate was virtually absent above 232 cm (*ca*. 5,450 ± 840 cal a BP). Above 144 cm (*ca*. 2,600 ± 130 cal a BP), BSi_diatoms_ increased from baseline concentrations and was 10% or more of sediment mass from 132 cm (*ca*. 2,260 ± 90 cal a BP) to the surface (Fig 6c). In contrast, BSi_sponges_ decreased up-core and the lowest concentration (<1%) was found in the surface sediments (Fig 6d).

Concentrations of H_2_O-P, HE-P and TP (Fig 4) in the sediment fluctuate considerably over the length of the core and do not display a common stratigraphic pattern. For all three variables, the range of concentrations far exceeded the upper bound for the data set (i.e. mean + 2σ). H_2_O-P ranged from 0.000 to 0.423 mg g^-1^ (0.073 + 0.100 mg g^-1^). The maximum in H_2_O-P concentration occurred in the middle Holocene at 236 cm (*ca*. 5,540 cal a BP). HE-P ranged from 0.000 to 0.311 mg g^-1^ (0.020 + 0.090 mg g^-1^). The maximum HE-P concentration was found in the 16-cm section (AD 1999 ± 1.8) and HE-P concentration was <0.01 mg g^-1^ in sediments below 320 cm. TP ranged from 0.216 to 1.534 mg g^-1^ (0.675 + 0.582 mg g^-1^). Maximum TP concentration was found in the 12-cm section (AD 2005 ± 1.7).

### Sediment accumulation rates

**Fig 7:**
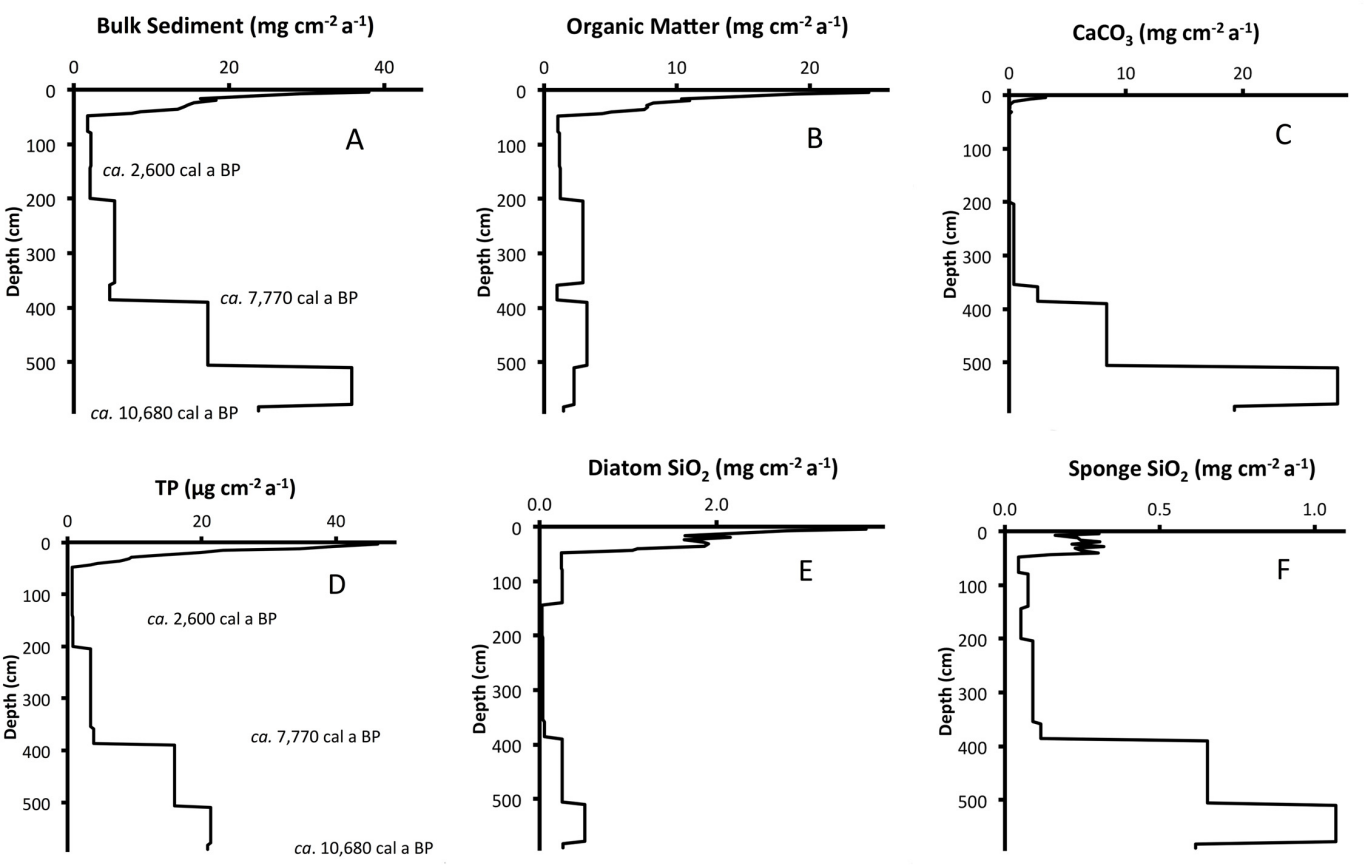
Mass Sedimentation Rates. Accumulation rates for (A) bulk sediment, (B) organic matter, (C) CaCO_3_, (D) total phosphorus, (E) diatom biogenic silica and (F) sponge spicule biogenic silica versus depth in core LH-6-13. The modeled basal age of the core is *ca*. 10,680 cal a BP. Bulk sediment accumulation rates indicate that the lake had filled enough to cover its modern surface area by *ca*. 7,770 cal a BP. Diatom biogenic silica concentrations and accumulation rates indicate that the lake had deepened to its modern limnetic state by *ca*. 2,600 cal a BP.

In LH-6-13, accumulation rates for the sediment variables decreased through much of the Holocene, remained relatively stable for several thousand years, and then increased in very recent times (Table 3, Fig 7). For OM, CaCO_3_, BSi_sponges_ and TP, MSRs stabilized at lowest values in the 198–44 cm section (*ca*. 4,500–40 cal a BP). The MSR for BSi_diatoms_ stabilized, with lowest values deeper in the sediments, 386–140 cm (*ca*. 7,770–2,500 cal a BP).

**Table 3:** Accumulation rates for sediment variables.

				Bulk Sediment			Organic Matter			CaCO_3_			Sponge Spicule			Diatom			Total Phosphorus	
Depth	Max Age	2σ Age Error	low	mean	high	low	mean	high	low	mean	high	low	mean	high	low	mean	high	low	mean	high
cm	a	a	mg cm^-2^ a^-1^	mg cm^-2^ a^-1^	mg cm^-2^ a^-1^	mg cm^-2^ a^-1^	mg cm^-2^ a^-1^	mg cm^-2^ a^-1^	mg cm^-2^ a^-1^	mg cm^-2^ a^-1^	mg cm^-2^ a^-1^	mg cm^-2^ a^-1^	mg cm^-2^ a^-1^	mg cm^-2^ a^-1^	mg cm^-2^ a^-1^	mg cm^-2^ a^-1^	mg cm^-2^ a^-1^	mg cm^-2^ a^-1^	mg cm^-2^ a^-1^	mg cm^-2^ a^-1^
0–44	102/39	8	13	14	15	7	8	9	0	0	0	0.2	0.2	0.3	1.5	1.6	1.8	11	11	12
44–78	922	62	2	2	2	1	1	1	0	0	0	0.0	0.0	0.0	0.2	0.2	0.3	1	1	1
78–140	2498	12	2	2	2	1	1	1	0	0	0	0.1	0.1	0.1	0.2	0.3	0.3	1	1	1
140–198	4489	76	2	2	2	1	1	1	0	0	0	0.1	0.1	0.1	0.0	0.0	0.0	1	1	1
198–354	6655	202	5	5	6	3	3	3	0	0	0	0.1	0.1	0.1	0.0	0.0	0.0	3	3	4
354–386	7770	42	4	5	6	1	1	1	2	2	3	0.1	0.1	0.1	0.0	0.1	0.1	3	4	5
386–504	9235	69	16	17	19	3	3	3	8	8	9	0.6	0.7	0.7	0.2	0.3	0.3	15	16	17
504–579	10362	69	32	36	41	2	2	3	25	28	32	1.0	1.1	1.2	0.5	0.5	0.6	19	21	24
579–590	10674	197	13	24	161	1	1	10	10	19	131	0.6	0.6	0.7	0.2	0.3	0.3	11	21	142

Accumulation rates for sediment variables for nine sediment intervals in core LH-6-13. Sponge spicule and diatom sedimentation rates are reported as SiO_2_. For each variable, the mean and 95% confidence interval bounds (low, high) are presented.

## Discussion

### Comparison with existing paleoecological framework for Florida

The chronology of stratigraphic changes in sediment characteristics for LH-6-13 is consistent with a paleoecological framework established from previous studies of Florida lakes. The modeled date for the basal sediments (*ca*. 10,680 cal a BP) indicates that the lake began to fill with water early in the Holocene. That timing is consistent with previous studies [12–15]. A shift from carbonate-rich (>50% CaCO_3_) to organic-rich sediment (>50% OM) at 354 cm (*ca*. 6,650 cal a BP) initiated a progression to sediments without measurable carbonate by 232 cm (*ca*. 5,440 cal a BP). The timing of this change in sediment characteristics coincides with the shift from oak- to pine-pollen predominance *ca*. 5,500 cal a BP in other Florida lake cores [13–19]. In Lake Harris, the concentration of BSi_diatoms_ increased abruptly above 144 cm (*ca*. 2,600 cal a BP). The increase in diatom concentration in Lake Harris sediments occurred about the same time as an increase in planktonic diatom abundance in the sediments from nearby Lake Apopka [12].

In LH-6-13, the deepest sediments, 590 to 514 cm (*ca*. 10,680 to 9,300 cal a BP), had the greatest carbonate content (>75% as CaCO_3_). High carbonate content of the sediments deposited at that time suggests that high-alkalinity aquifer groundwater was an important component of the lake’s hydrologic budget. These sediments (590–514 cm) also had BSi_sponges_ concentrations, more than double that of BSi_diatoms_. This contrasts with the late Holocene condition of the lake that shows ~5-fold more BSi_diatoms_ than BSi_sponges_. Comparison of late Holocene BSi_sponges_:BSi_diatoms_ to initial BSi_sponges_:BSi_diatoms_ indicates that modern Lake Harris is deeper than it was in the early Holocene. The carbonate content and BSi_sponges_:BSi_diatoms_ of the deepest sediments from Lake Harris suggest a shallow system with high-alkalinity water and ample substrate for freshwater sponges. Nearby Lake Panasoffkee [33], which today receives substantial inputs of high-alkalinity water from the Floridan Aquifer, may provide a modern analog for the early Holocene state of Lake Harris, because it is shallow, produces carbonate sediments and is dominated by submersed macrophytes. Donar et al. [12] inferred a similar condition for the early stages of nearby Lake Apopka. There, deepest sediments contained a benthic-alkalibiontic diatom flora indicative of a shallow fen with high-alkalinity water [12].

We used the carbonate content of sediments as a proxy for relative aquifer-groundwater contributions to the lake. Thus, the decrease in carbonate content of sediments from *ca*. 6,650 to *ca*. 5,450 cal a BP signals a reduction in the proportional contribution of aquifer-groundwater inputs to the lake relative to rainfall and surface runoff received by the lake. A reduction in groundwater inputs with lake age was attributed to Holocene soil development around boreal and temperate lakes of North America [2], [3]. In post-glacial landscapes, the accumulation of OM in the soil profile diminished hydrologic recharge of groundwater, increased surface runoff and provided organic acids to consume alkalinity [2], [3]. Although the Florida landscape was not glaciated [15], it is reasonable to assume that Holocene soil development in Florida provided organic acids to consume some of the alkalinity of groundwater inputs to Lake Harris. But in Florida, increased rainfall also had an influence on the hydrologic budgets of shallow lakes. A mid-Holocene shift to a wetter climate in Florida decreased the relative proportion of aquifer-groundwater inputs to Lake Harris by increasing the relative proportion of rainfall and surface runoff inputs to the lake. Numerous studies of Holocene pollen stratigraphy in Florida lakes [13–19] reported a shift from predominantly oak to pine pollen *ca*. 5,500 cal a BP, which was attributed to the onset of wetter climate. This vegetation shift is coincident with the decrease in carbonate content we found in LH-6-13 from 354 to 232 cm, and also indicates increased rainfall in the Lake Harris watershed.

Similar to Donar et al. [12], we used the diatom contribution to sediments as a proxy for lake depth. In Lake Apopka, a shift from a greater proportion of benthic diatoms (>50%) to a greater proportion of planktonic diatoms (>50%) *ca*. 2,800 cal a BP indicated that the system had increased in depth sufficiently to approximate its modern limnetic state [12]. In lieu of taxonomic diatom data from LH-6-13, we used concentrations and accumulation rates of biogenic silica fractions to infer past changes in water depth. In Lake Harris, we observed an increase in BSi_diatoms_ concentration and accumulation rate *ca*. 2,600 cal a BP. The sediments deposited at that time also recorded a shift to a greater proportion of BSi_diatoms_ (>50%) than BSi_sponges_ (<50%). Because these changes in biogenic silica stratigraphy from Lake Harris did not coincide with increased TP concentrations or accumulation rates, they could not be attributed to eutrophication, so it is likely that these shifts in biogenic silica sedimentation resulted from increased water depth. Therefore, sediment records from the two lakes indicate that the modern limnetic states were achieved in Lake Apopka (*ca*. 2,800 cal a BP) and Lake Harris (*ca*. 2,600 cal a BP) at nearly the same time. The slightly older age for this development in Lake Apopka may be explained by the fact that ^14^C ages for the Lake Apopka core were measured on bulk sediments and not corrected for hard-water-lake error. If the dating offset for hard-water error in Lake Apopka sediments were similar to that measured in Lake Harris, then the timing of the development of the modern limnetic state is even closer for the two lakes.

### Accumulation rates as proxies for trophic state trajectory

In LH-6-13, an up-core decrease in MSR for sediment variables OM, CaCO_3_, TP, BSi_sponges_ and BSi_diatoms_ indicates time-course oligotrophication of the lake through the Holocene. In sediments above 44 cm, increased MSR for variables OM, CaCO_3_, TP, BSi_sponges_ and BSi_diatoms_ indicates progressive cultural eutrophication after *ca*. AD 1900. BSi_diatoms_-MSR increased above minimum values *ca*. 2,600 cal a BP, but there was no coincident increase in TP-MSR; therefore the late-Holocene increase in BSi_diatoms_-MSR is not attributed to eutrophication, but rather to increased lake depth. It is possible that the observed up-core decrease in sedimentation rates was caused by a sediment hiatus at the coring location. It is, however, unlikely that LH-6 was an erosional site in the past because the location falls within the 20% of the lakebed that possesses the greatest soft sediment thickness (>5.9 m). Also, the chronological models (age-depth, age-cumulative mass) have high r^2^ values (>0.95), suggesting uniform, predictable sedimentation over time at this location. A sediment hiatus would likely be caused by a stochastic (i.e. unpredictable) event and result in poor model performance for predicting age from depth or cumulative mass. Finally, sediment variables in the sections with the lowest MSRs were reasonably consistent and did not suggest that part of the record was missing.

It is possible that the observed up-core decrease in sedimentation rate resulted from a decrease in sediment focusing as the lake filled and the area of the lakebed that accumulated sediments increased. The potential for temporal changes in sediment focusing to obscure the interpretation of calculated Holocene sedimentation rates was demonstrated in Mirror Lake, New Hampshire, USA [34]. Because LH-6-99 site is within the upper quintile of the lakebed with respect to soft sediment thickness, it is likely that accumulation rates calculated for the deepest sediments are influenced by sediment focusing that occurred before the lake filled to cover its much larger modern surface area. For LH-6-99, bulk sediment-MSR decreased up-core to a relative minimum at 386 cm (*ca*. 7,770 cal a BP). We consider this trend of decreasing bulk sediment-MSR during the early phase of the lake, i.e. before *ca*. 7,770 cal a BP, to have been a consequence of decreased sediment focusing as the lake filled and the surface area of the lakebed increased. Above this depth (386 cm), up to 44 cm, bulk sediment-MSR was lower (<5.3 mg cm^-2^ a) than that found in deeper sediments (>17 mg cm^-2^ a) of the early Holocene, or in the sediments above 44 cm (7.5–38.0 mg cm^-2^ a). Because of the relatively low and stable bulk sediment-MSR over the time period from *ca*. 7,770 cal a BP to AD 1900, we conclude that the lake had filled to cover its modern surface area by *ca*. 7,770 cal a BP, and after that time sediment focusing had a lesser influence on sedimentation rates at LH-6 than it had earlier in the sediment record.

Considering the sediments deposited above 386 cm, i.e. after *ca*. 7,770 cal a BP, accumulation rates of sediment variables TP-MSR, OM-MSR and bulk sediment-MSR indicate oligotrophication coincident with primary succession in Lake Harris. OM-MSR and bulk sediment-MSR both showed an almost 3-fold decrease over the period from *ca*. 7,770 cal a BP to AD 1900 and TP-MSR decreased >5-fold during that time. The pattern of time-course oligotrophication coincident with primary succession in Lake Harris is consistent with studies of boreal lakes [2] and temperate lakes [3] in North America.

We invoke a combination of external and internal factors to explain the observed decrease in TP-MSR through the Holocene record of LH-6-13. External factors included decreased P-loading from groundwater and increased P assimilation by terrestrial vegetation in the Lake Harris catchment. Localized external factors such as vegetation history and soil development were shown to exert primary control over nutrient concentrations in boreal lakes of North America [35]. As the climate shifted to wetter conditions, i.e. greater precipitation through the Holocene, the corresponding increase in terrestrial vegetation assimilated more atmospherically loaded P, which decreased the P load to the lake from the watershed. Decreased P retention in the sediments, associated with development of a lake outflow, is an internal factor that could have also contributed to decreased TP-MSR. The magnitude of P retention in the sediments is inversely proportional to hydrological outflow [36]. As Lake Harris filled, it is reasonable to assume that the lake shifted from a closed system with no outflows, to a system with intermediate volume outflows and finally to the modern system that is hydrologically connected to downstream Lake Eustis. As lake stage increased and hydrologic outflows developed and their flow increased, P was increasingly lost to downstream systems and P retention in sediments, i.e. sedimentation/loading, decreased. These internal and external factors worked in combination to decrease TP-MSR in the lake throughout most of the Holocene.

The trophic state trajectory of oligotrophication throughout most of the late Holocene contrasts sharply with the trophic state trajectory of eutrophication inferred from sediments deposited in the last ~100 years. The record of bulk sediment, OM and TP accumulation rates from LH-6-13, suggests that before European settlement, the lake was asymptotically approaching equilibrium with terrestrial inputs from the watershed. In the absence of anthropogenic alterations to the watershed and cultural eutrophication, it seems likely that sedimentation rates would have continued to decline to even lower values than those calculated for much of the late Holocene. This suggests that comparisons of modern conditions in the lake to pre-disturbance conditions in the lake (i.e. immediately pre-1900), underestimate the true impact of cultural eutrophication.

### Phosphorus sedimentation and post-depositional mobility

Because of concerns over post-depositional P mobility, some investigators have questioned the utility of sediment P fractions as proxies for eutrophication [37], [38]. We argue, however, that the complex and very different stratigraphies of H_2_O-P, HE-P and TP, as well as discrete peaks in concentration of these variables in LH-6-13 demonstrate a lack of post-depositional mobility for these P forms in the Lake Harris core. The sediment records of P fractions in LH-6-13 vary greatly with depth and lack the “smoothing” one might expect over the course of thousands of years if concentration gradients were reduced by diffusive flux of P. Such “smoothing” would have occurred as P moved from depths with higher concentrations to adjacent sections with lower concentrations. If P mobility were significant, then given sufficient time, sediment P concentrations should equilibrate to an average value, minimizing or eliminating concentration gradients along the length of the core.

The H_2_O-P sediment fraction should be the most subject to post-depositional mobility of the P fractions measured here, because it is water-soluble, bioavailable and chemically reactive [28]. The H_2_O-P record shows that 3.4% of the sediment sections had greater concentrations than the upper bound of the data set (i.e. mean + 2σ) and 97% of the samples fall outside of the 95% confidence interval for the mean (i.e. mean ± 2 s.e.). One example that argues strongly against post-depositional mobility comes from the 236-232-cm section (*ca*. 5,540 cal a BP) and the sediments immediately above and below it. In the interval from 236 to 232 cm, the H_2_O-P concentration is 0.423 mg g^-1^, but the adjacent 4-cm samples had only 0.083 and 0.157 mg g^-1^. Thus, a substantial concentration gradient was maintained over a short depth increment for thousands of years. Furthermore, the relatively broad sampling interval over which H_2_O-P was measured (4-cm) likely was responsible for some “smoothing” of the record. Despite having been deposited >5 ka ago, there is no evidence for post-depositional mobility of the most mobile P fraction in these sediments. There are similar examples elsewhere in the H_2_O-P profile and in the profiles of HE-P and TP. Sediment records of P fractions from LH-6-13 have not been “smoothed” by post-depositional mobility, and we are therefore confident about using the P fractions as reliable proxies for P delivery and lake eutrophication.

Like H_2_O-P, the HE-P stratigraphy displays numerous concentration “peaks” (i.e. localized relative maxima) in sediments older than *ca*. 2,500 cal a BP. Kenney et al. [28] referred to the HE-P fraction as equivalent to polyphosphate (Poly-P), but HE-P may also include other organic components of sediment P [38], [39]. To avoid confusion, we use the operationally defined term HE-P here. Because the HE-P fraction may include Poly-P in intact, sedimented phytoplankton [40] and HE-P was shown to be correlated strongly to phytoplankton indicators such as native chlorophyll [41] and diatom biogenic silica [40], we consider the down-core peaks of HE-P to be indicators of past periods of high phytoplankton deposition. Presence of HE-P in Lake Harris sediment that is thousands of years old (*ca*. 6,000–2,600 cal a BP) suggests that this P fraction has components that are stable and not subject to post-depositional mobility. We infer that Lake Harris had filled to cover its modern surface area by *ca*. 7,770 cal a BP and had deepened to its modern limnetic state by *ca*. 2,600 cal a BP. Presence of HE-P peaks in sediments deposited from *ca*. 7,770 to 2,600 cal a BP indicates the system was functioning as an open-water lake at that time.

## Conclusions

We used geochemical data from a 5.9-m sediment core (LH-6-13) taken in shallow Lake Harris, FL, USA to gain insights into the historical development of the lake and its current eutrophic status. The chronology of the core was established using ^210^Pb and ^14^C dating. Stratigraphic changes in sediment variables for LH-6-13 are consistent with a paleoecological framework established from core studies in several other Florida lakes. Lake Harris began to fill in the early Holocene, *ca*. 10,680 cal a BP. A shift from carbonate to organic sediments indicated the transition to a wetter climate in the middle Holocene, *ca*. 5,540 cal a BP. A rapid increase in diatom biogenic silica concentrations and accumulation rates *ca*. 2,600 cal a BP signaled that the lake had deepened to its modern limnetic state. In LH-6-13, an up-core decrease in accumulation rates for many sediment variables indicated time-course oligotrophication of the lake through the Holocene. In sediments above 44 cm, increased sedimentation rates for several variables indicate progressive cultural eutrophication after *ca*. AD 1900. Comparison of the modern state of Lake Harris to its condition 50 or 100 years ago provides some measure of the impact of cultural eutrophication. If, however, we consider that the pre-disturbance trajectory of this lake was one of oligotrophication, then we conclude that the true impact of cultural eutrophication is much greater than that inferred from the changes that occurred in the recent past.

## Supporting Information

S1 Table Sediment Core Data. (XLSX)
